# Behavioural, immunological and transcriptomic consequences of post-weaning social isolation and chronic celecoxib administration in mouse

**DOI:** 10.1371/journal.pone.0334451

**Published:** 2025-10-22

**Authors:** Aodán Laighneach, Derek W. Morris, Saahithh Redddi Patlola, Marcelo Improta, Lieve Desbonnet, Gary Donohoe, John P. Kelly, Declan P. McKernan

**Affiliations:** 1 Centre for Neuroimaging, Cognition and Genomics, Galway Neuroscience Centre, University of Galway, Galway, Ireland; 2 School of Biological and Chemical Sciences, University of Galway, Galway, Ireland; 3 Discipline of Pharmacology & Therapeutics, School of Medicine, University of Galway, Galway, Ireland; 4 School of Psychology, University of Galway, Galway, Ireland; University of California Santa Barbara, UNITED STATES OF AMERICA

## Abstract

Early life stress (ELS) and chronic low-grade inflammation are associated with psychiatric disease risk, but their neurobiological consequences are poorly understood. Here, we aim to investigate the behavioural, immunological and molecular consequences of ELS in mice. C57Bl6 mice were subjected to post-weaning social isolation (SI - PD21−40) with or without chronic celecoxib (CEL) (PD21−61). ELS-induced behavioural changes were assessed using the open field test (OFT) and three-chambered test (3CT). The anti-inflammatory effects of celecoxib were assessed by enzyme-linked immunosorbent assay (ELISA) of IL-6, TNF-α and IL-10 cytokines released by stimulated splenocytes. Gene expression changes in the hippocampus and amygdala were assessed using RNA-sequencing. Neither SI nor CEL affected OFT time in centre or 3CT discrimination ratio. However, SI induced locomotor changes in both tests. CEL significantly reduced IL-6, TNF-α and IL-10 release from splenocytes. SI induced significant gene expression changes in both hippocampus and amygdala, while CEL only induced gene expression changes in the hippocampus. Differentially expressed genes (DEGs) induced by SI were enriched for ontologies relating to gamma-aminobutyric acid activity and insulin binding in the hippocampus and neurogenesis in the amygdala. CEL-induced DEGs in the hippocampus were enriched for neurogenesis. Cell type enrichment implicated choroid plexus and vascular leptomeningeal cells in SI DEGs and medium spiny neurons (MSNs) in CEL DEGs. CEL-induced DEGs were enriched for heritability for psychiatric disorders and cognitive ability. In conclusion, gene expression changes show convergence with human psychiatric disorders through both enrichments in common genetic heritability and enrichment of previously implicated cell populations.

## Introduction

The course of human brain development is strongly dependant on genetic and environmental factors. Psychiatric disorders including schizophrenia (SCZ) [1,2], bipolar disorder (BPD) [3] and major depressive disorder (MDD) [4] are often considered to have a neurodevelopmental basis, in which a combination of genetic liability and early life stressors offset a normal neurodevelopmental trajectory. Although the contribution of human genetic risk factors for these disorders is well investigated by large genome-wide association studies (GWAS) [5–7], investigating environmental risk factors in humans is much more difficult and are generally restricted to observational work. Animal models provide a feasible alternative to study potential stressors.

Early life stress in humans can impact developmental trajectory and maturation of the brain [8] leading to detrimental changes in physical [9] and mental health [10]. Social isolation (SI) is a form of stress in which an individual is separated from society or normal social interaction. Being exposed to SI leads to loneliness and can contribute to increased risk of some psychiatric disorders and related traits, including anxiety and depression [11,12], SCZ [13] and cognitive decline [14]. Furthermore, in addition to SI contributing to disorders, individuals with severe mental illness can be vulnerable to regressing into a socially isolated environment, such as social withdrawal seen in SCZ [15]. Another factor linked with neuropsychiatric disorders is underlying inflammation [16]. Low-grade chronic systemic inflammation as indicated by elevations in pro-inflammatory cytokines, through interaction with neuronal immune molecules, stress regulation pathways and glial cell processes have been hypothesised to alter the trajectory of neurodevelopment [17]. Altering the levels of cytokines through the use of non-steroidal anti-inflammatory drugs (NSAIDs), such as celecoxib (CEL), could give an insight into the role of inflammation in brain responses to chronic stress. In addition, celecoxib has been used as an adjunct therapy in depression [18], anxiety [19] and even early stage schizophrenia [20,21].

Large-scale studies of gene expression involving RNA-seq, facilitate an investigation into the molecular underpinnings of environmental stressors on mouse models. Such analyses of brain tissue of animals exposed to SI or CEL are rarer compared to behavioural experiments. Previous work by our group [22] showed SI leads to changes in hippocampal gene expression relating to synapse structure and function. Similar work [23] in mouse also showed changes in synapse biology, as well as ion channel function, limbic system development and neuropeptide signalling. Transcriptomic work using microarray in rat cortex [24] found dysregulation of genes related to synaptic structure and function, notably inhibitory GABAergic synapses. No RNA-seq or microarray analysis has focused on the interaction of SI and CEL on gene expression in brain regions. However, microarray analysis of the rat hippocampus using another NSAID, indomethacin, [25] identified a small number of differentially expressed genes (DEGs; 14 upregulated, 6 downregulated), including the glucocorticoid-inducible SGK1, which has a role in the stress response and hippocampal neurogenesis [26] and NPY, which regulates stress, anxiety and depression [27].

Here, we build on previous work by our group investigating the behavioural [28] and transcriptomic [22] effects of SI. We aimed to investigate the impact of SI housing and effects of CEL administration in mice. We hypothesised that social isolation would induce behavioural and inflammatory changes in mice and there would be an interaction between SI and CEL. Our study presents a valuable insight into the behavioural, immunological and transcriptomic effects of these factors in isolation. We investigate gene expression in hippocampus and amygdala – regions involved in relaying and integrating environmental messages of fear and anxiety [29]. Furthermore, we investigate how these changes compare to our previous SI work as well assess how they relate to synapse biology, how they relate to known markers of specific cell types and how they converge with the genetic basis of human neurodevelopmental and neuropsychiatric disorders.

## Methods

### Ethics statement

All procedures received ethical approval from the local Animal Care Research Ethics Committee (ACREC) and the Health Products Regulatory Authority in Ireland (licence number AE19125/P110).

### Animals and treatment procedures

Pregnant C57BL/6 mice were obtained from Charles River Laboratories (UK). Dams were then allowed to litter, and the number of offspring were counted and reduced where necessary to 8–10 pups for each litter. Upon weaning at PND 21, female mice were randomly allocated either to be housed in groups of 4 animals per cage (Group-Housed, GH) or socially isolated (SI) in the same holding room until sacrifice. On PD43, animals received either 30 mg/kg celecoxib (CEL) or a 1:1 dilution of water:condensed milk as vehicle control (Veh) via oral micropipette-guided drug administration (MDA) [30]. This led to a final four groups as follows: SI-Veh (n = 8); SI-CEL (n = 8); GH-CEL (n = 8) and GH-Veh (n = 8). Animals underwent behavioural tests on PD 57 and PD60 and were sacrificed via decapitation following carbon dioxide asphyxiation on PD61. Spleens were dissected under sterile conditions and splenocytes were cultured as described below. Whole brain was removed and snap frozen on dry ice. Hippocampus and amygdala were later dissected on ice and stored at − 80 °C until RNA extraction as described below.

### Open field test

The open field test is used to assess emotionality and anxiety-related behaviour in mice [31]. Mice are positioned in a square open field (opaque white acrylic box; 40 x 40 x 30 cm) in a brightly lit room (74 lux) for a period of 10 min. The distance moved, movement velocity, and time spent in the center zone (20 x 20 cm center square in each arena) and corner zone (10 x 10 cm zone in each corner of the arena) was measured from recorded videos by automated tracking (EthoVision, Version 14, TrackSys Ltd., UK). The OFT took place on PD57.

### Three-chambered sociability and social memory test

Mice were placed into a rectangular container (36 x 20 x 20 cm) with three chambers (left and right chambers, 13.5 x 20 x 20 cm; centre chamber, 9 x 20 x 20 cm) divided by transparent partitions with small circular openings providing access to all compartments. The test comprised of three 10 min trials in the following sequence: (1) habituation (the mouse was free to explore the empty chambers); (2) sociability (an unfamiliar mouse was placed in an inner wire mesh cage in either the left or right chambers; time spent in the chamber with the mouse versus the empty chamber is recorded as a measure of sociability); (3) social memory (a stranger mouse is placed into the empty inner cage in the chamber opposite the now familiar mouse; time spent in the chamber with the familiar mouse vs the chamber with the novel mouse is recorded). Mouse behaviour was recorded by a ceiling-mounted video camera and time spent in each of the chambers and total distance moved over the three trials was calculated using automated EthoVision video tracking software to determine sociability and social memory. The 3CT took place on PD 60.

### Splenocyte cytokine response

Splenocyte cytokine responses to TLR2 [Heat Killed Listeria monocytogenes (HKLM); InvivoGen, France; product code, tlrl-hklm], TLR3 (PIC; InvivoGen, France; product code, tlrl-pic) and TLR4 ligands [lipopolysaccharide (LPS); InvivoGen, France; product code, tlrl-eklps]. Spleens were dissected under sterile conditions, weighed and mashed through a 70 µM sterile sieve and suspended in 5 ml sterile RPMI culture medium (Sigma-Aldrich Ireland, product code: R8758–6X500ML) heated to 37 °C. Splenocytes (500 µL) from each sample were quantified and samples were normalised to a concentration of 5 × 10^5^ cells/mL. Each TLR ligand (Final concentration – LPS 1 µg/ml, HKLM 10^8 cells, PIC 10 µg/ml) was added to 500ul of splenocyte sample (1:100 dilution with RPMI) and each sample was assayed in duplicate in a sterile flat-bottomed 24-well plate and incubated for 24 h (37 °C, 5% CO2). After 24 h, plates were centrifuged (1300 rpm, 5 min, 21ºC) and cell culture supernatants were collected into sterile tubes and stored at − 80 °C until assayed for IL6, TNF-α and IL10 cytokines concentrations using IL-6, TNF-α and IL10 ELISA Duoset, and Ancillary Reagent kits (R&D Systems, product codes: DY406, DY008 & DY417) according to manufacturers’ instructions.

### RNA extraction and sequencing

Tissue was processed for 64 samples (32 hippocampus and 32 amygdala from each animal) from the above behavioural study and proceeded as follows: Total RNA was extracted from frozen hippocampus and amygdala using the Absolutely RNA Miniprep kit (Agilent; product code: #400800) following appropriate standard procedure for quantity of tissue. Once RNA yield (> 500 ng), purity and integrity (RIN > 6) was confirmed, RNA-seq was performed on the Illumina NovaSeq (paired-end; 2x150 bp reads) sequencing platform producing a minimum of> 20M reads/sample (Genewiz Germany GmbH). Four samples were excluded based technical QC factors.

### Differential expression analysis

Raw data was received through SFTP in FASTQC format. *Trimmomatic* v0.39 [32] was used to remove low quality and adapter sequences from the paired-end reads (LEADING:3, TRAILING:3, MINLEN:36). *Salmon* v1.8.0 [33] was used to quasi-map to the *mm39* transcriptome and quantify reads. The *DEseq2* v1.24.0 [34] R package was used to test genes for differential expression. To account for batch effects, date of extraction was used as a covariate in the DE analysis. DEGs were defined at a false discovery rate (FDR) of < 0.05. Genes were converted to human orthologues using biomaRt v2.40.5 [35] where necessary.

### Gene ontology analysis

Gene ontology (GO) analysis was done using ConsensusPathDB (http://cpdb.molgen.mpg.de/) [36] over-representation analysis. Ontologies with GO term levels 2–5 were tested and biological processes (BPs), cellular compartments (CCs) and Molecular functions (MFs) with an FDR-corrected p-value < 0.05 were considered significantly enriched.

### Cell type enrichment analysis

Data from single cell RNA-seq (scRNA-seq) of the mouse nervous system [37] was used to test if different cell types were enriched for DEGs. Analysis was performed using the expression-weighted cell type enrichment (EWCE) R package [38], which investigated whether the cell types were significantly enriched for a gene-set when weighted by gene expression. Cell types were considered significantly enriched at a Bonferroni-corrected p-value of < 0.05 (correcting for the number of cell types tested).

### Gene enrichment analysis associated with neurodevelopmental phenotypes

Data on common variants (SNPs) associated with human phenotypes were accessed in the form of GWAS summary stats for a range of phenotypes relevant to neurodevelopment and psychiatric disorders including SCZ (GWAS based on 67,390 cases and 94,015 controls) [5], intelligence (IQ; 269,867 individuals) [39] educational attainment (EA; 766,345 individuals) [40], bipolar disorder (BPD; 41,917 cases and 371,549 controls) [6], major depressive disorder (MDD; 246,636 cases and 561,190 controls) [7]. As control phenotypes, GWAS data for attention deficit hyperactivity disorder (ADHD; 20,183 cases and 35,191 controls) [41], Alzheimer’s disease (AlzD; 71,880 cases and 383,378 controls) [42], stroke (40,585 cases and 406,111 controls) [43], type-2 diabetes (T2D; 74,124 cases and 824,006 controls) [44] and coronary artery disease (CAD; 22,233 cases and 64,762 controls) [45] were used. Stratified LD Score regression (sLDSC) was used to investigate if gene-sets were significantly enriched for SNP heritability contributing to the test and control phenotypes [46,47]. Gene start and stop coordinates of each DEG on GRCh37 were found using biomaRt v2.40.5 [35]. Annotation files were generated for each chromosome in each set of DEGs using 1000 Genomes European cohort SNPs and a window size of 100kb extended to coordinates [48]. LD scores were estimated within a 1cM window using 1000 Genomes Phase 3 European reference panel. Heritability was stratified in a joint analysis between 53 previous function genomic annotations [47] and each set of DEGs. Only SNPs from HapMap Project phase 3 SNPs with a MAF > 0.05 were considered in this analysis.

### Statistics

**Fig 1:**
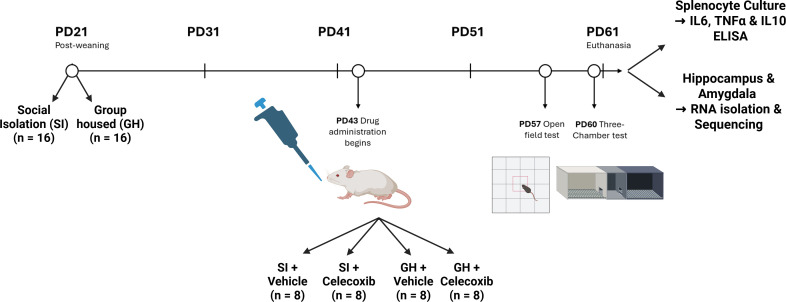
Timeline of behavioural animal work. Animals began post-weaning SI at PD21 and began drug treatment at PD43. Behavioural assays were conducted on PD57 and PD60. Animals were sacrificed on PD61. Created in BioRender. https://BioRender.com/h94q048.

**Fig 2:**
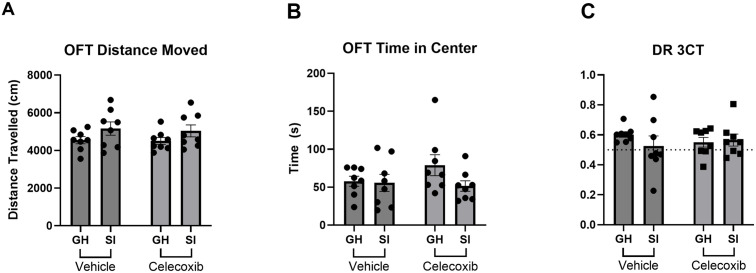
Behavioural outcomes of SI and CEL exposure. The effect of SI and CEL exposure on anxiety-like and social behaviours were measured. A) Open field test (OFT) distance moved B) OFT Time in center C) Three-chambered test (3CT) discrimination Ratio (DR). DR of 0.5 (no discrimination) shown as dashed line. Data are expressed as mean ± SEM.

**Fig 3:**
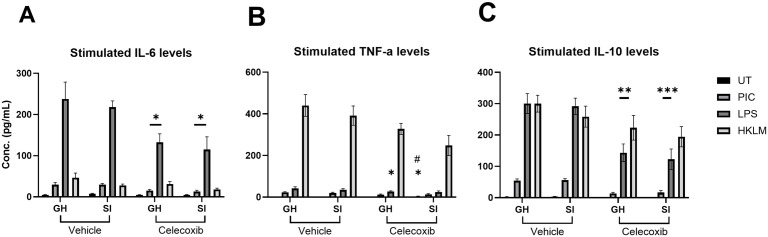
Stimulated cytokine levels. TLR-stimulated levels of inflammatory cytokines A) IL6 B) TNFa and C) IL10 in cultured splenocytes. * = p < 0.05; ** = p < 0.01 and *** = p, 0.001 compared to vehicle-treated control # = p < 0.05 compared to group-houses control in Sidak’s post-hoc tests. Data are expressed as mean ± SEM.

The effect SI and CEL exposure on behavioural outcomes in the open field and 3-chamber tests was analysed using 2-way ANOVA (Drug x Housing) in GraphPad Prism 10.2.0. Cytokine concentrations from ELISA standard curves were interpolated using a cubic spline and the main effects were analysed using 2-way ANOVA (Drug x Housing) GraphPad Prism 10.2.0. Normality of residuals was assessed using the Shapiro-Wilk test and equal variance was assessed using Spearman’s test for heteroscedasticity. Graphical representations in Figs 1, 2 and 3 are provided as mean ± SEM.

## Results

### Behavioural outcomes associated with SI and CEL exposure

In order to confirm the impact of SI on mouse behaviour seen in our previous work [28], the OFT (anxiety) and the 3CT (sociability) behaviours were assessed in all treatment groups. Mice exposed to SI showed an overall increase of 12.6% in total distance moved [SI effect: F(1,28) = 4.443, p < 0.05] (Fig 2A) and average movement velocity [SI effect: F(1,28) = 4.443, p < 0.05] (S1 Fig). Although there was an average decrease of 21.6% of time in centre in socially-isolated animals, this was not statistically significant (Fig 2B). There were no behavioural effects of CEL administration or interaction effects [SI x CEL] present in the open field test. Neither exposure to SI housing nor CEL administration had a significant impact on discrimination ratio between novel and familiar mice in the 3CT social memory trial (Fig 2C). Exposure to SI housing led to a 12.6% decrease in locomotor activity in the sociability phase [SI effect: F (1, 28) = 11.33, P < 0.01] and 14.1% decreased in locomotor activity in the social memory phase [SI effect: F (1, 28) = 13.72, P < 0.001], but no effect in habituation phase of the 3CT (S2 Fig).

### Immunological outcomes associated with SI and CEL exposure

In order to confirm the efficacy and dose of CEL *in vivo*, we tested the effects on *ex vivo* stimulated splenocytes with agonists of TLR1/2 (HKLM), TLR3 (PIC) and TLR4 (LPS). Animals subject to chronic CEL administration showed decreased IL-6 (Fig 3A) response to stimulations from PIC [CEL effect: F (1, 28) = 15.52, P < 0.001] and LPS [CEL effect: F (1, 28) = 13.14, P < 0.01], but not HKLM or basal IL-6 levels in UT control, compared to vehicle-treated animals. Animals exposed to SI housing showed a decreased response to HKLM stimulation [ F (1, 28) = 5.435, P = 0.0272], but not PIC, LPS or basal IL-6 levels in UT control, compared to group-housed animals. No interaction (SI x CEL) effect was found in IL-6 response to TLR stimulation. CEL administration also led to decreased TNF-α response (Fig 3B) to stimulation with PIC [CEL effect: F (1, 28) = 7.917, P < 0.01], LPS [CEL effect: F (1, 28) = 5.005, P < 0.05] and HKLM [CEL effect: F (1, 28) = 8.220, P < 0.01] conditions, but not basal TNF-α levels in UT control. There were no effects of SI housing or interaction on the TNF-α response to any stimulation condition. Finally, administration of CEL led to a decreased IL-10 response (Fig 3C) from stimulation with PIC [CEL effect: F (1, 27) = 60.91, P < 0.0001], LPS [CEL effect: F (1, 28) = 29.59, P < 0.0001], HKLM [CEL effect: F (1, 28) = 4.488, P < 0.05] and basal IL10 levels in UT control [CEL effect: F (1, 28) = 7.744, P < 0.01]. SI and CEL factors induced.

### Transcriptomic outcomes associated with SI and CEL exposure

#### Differential expression analysis.

**Fig 4:**
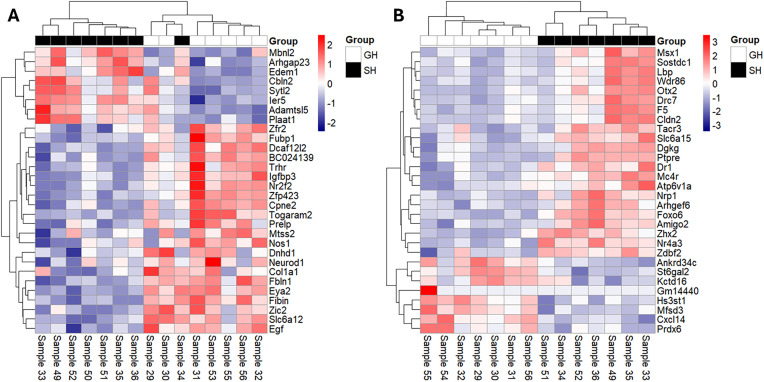
Heatmap of top 30 SI-induced gene expression changes by p-value. Normalised gene expression changes of the top 30 SI-induced gene expression changes in (A) hippocampus and (B) amygdala. Expression values were variance-stabilizing transformed (VST) and mean-centered across samples. Red = Increase in gene expression in single-housed (SH) vs group-housed (GH); blue = decrease; white = no change.

**Fig 5:**
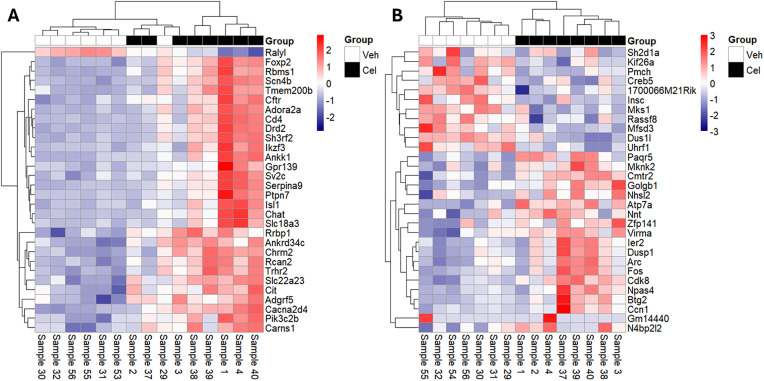
Heatmap of top 30 CEL-induced gene expression changes by p-value. Normalised gene expression changes of the top 30 CEL-induced gene expression changes in (A) hippocampus and (B) amygdala. Expression values were variance-stabilizing transformed (VST) and mean-centered across samples. Red = Increase in gene expression in celecoxib-treated (Cel) vs vehicle-treated (Veh); blue = decrease; white = no change. Note: no DEGs survived FDR-correction in the amygdala.

To assess the molecular effects in the brain induced by SI and CEL, gene expression changes in the hippocampus and amygdala were investigated using RNA-seq. The top 30 genes by p-value dysregulated by SI (Fig 4) and CEL (Fig 5) with their magnitude and direction are illustrated below. SI induced at total of 55 and 137 DEGs at an FDR < 0.05 in hippocampus and amygdala respectively. For hippocampus, around two thirds (63%) were downregulated while for the amygdala less than one third (29%) was downregulated.

CEL administration induced 355 DEGs at an FDR < 0.05 in the hippocampus. Almost four fifths of these (79%) were upregulated. No DEGs were detected at FDR < 0.05 for the effect of CEL in the amygdala. No DEGs were detected in either region based on both factors (SI + CEL) being present. Among the DEGs induced by SI, one gene, *Igfbp3*, an insulin like growth factor binding protein was common between both brain regions. In the hippocampus, where both individual SI and CEL factors induced gene expression changes, five genes (*Adamtsl5*, *Cdh6*, *Hrasls/Plaat1*, *Kcnab3* and *Neurod1*) were dysregulated by both SI and CEL. Full gene expression results can be found in Supplementary Table 1 in S1 Tables.

#### Gene ontology analysis.

Gene ontology analysis was performed to add biological context to gene expression changes in these models. Table 1 below show the top level 4 or 5 overrepresented GO terms by Q-value for SI and CEL-induced DEGs respectively. Among the top overrepresented ontologies for SI-induced hippocampal DEGs were molecular functions specific to neurotransmitter signalling and ion transport including gamma-aminobutyric acid:sodium symporter activity (GO:0005332; FDR adjusted P (or Q) = 0.0012) and calcium ion binding (GO:0005509; Q = 0.0020). The most overrepresented biological processes for these DEGs were epithelial cell proliferation (GO:0050673; Q = 0.0043), head development (GO:0060322; Q = 0.019) and cell proliferation in hindbrain (GO:0021534; Q = 0.024). Most of the top overrepresented ontologies for amygdala FDR-significant DEGs were biological processes with generation of neurons (GO:0048699; Q = 0.00013), cell differentiation (GO:0030154; Q = 0.00016), regulation of localization (GO:0032879; Q = 0.00017) and neuron projection guidance (GO:0097485; Q = 0.00017). Four GO terms were overrepresented in both hippocampal and amygdala DEG sets: growth factor binding (GO:0019838), head development (GO:0060322), type B pancreatic cell proliferation (GO:0044342) and brain development (GO:0007420).

**Table 1:** Top 5 overrepresented GO terms with level 4/5 term level from ConsensusPathDB within SI and CEL-induced DEGs.

Factor	Tissue	GO Term	Name	GO Level	FDR Q Value
SI	**Hipp.**	GO:0005332	gamma-aminobutyric acid:sodium symporter activity	4	0.001241
		GO:0005509	calcium ion binding	5	0.002027
		GO:0031995	insulin-like growth factor II binding	5	0.00232
		GO:0140161	monocarboxylate:sodium symporter activity	5	0.002563
		GO:0031994	insulin-like growth factor I binding	5	0.002563
	**Amy.**	GO:0048699	generation of neurons	5	0.00013
		GO:0060259	regulation of feeding behavior	4	0.000234
		GO:0022008	neurogenesis	4	0.000234
		GO:0007399	nervous system development	4	0.000234
		GO:0007411	axon guidance	4	0.000234
CEL	**Hipp.**	GO:0043005	neuron projection	4	1.40E-09
		GO:0030424	axon	4	5.70E-09
		GO:0007399	nervous system development	4	1.23E-07
		GO:0022008	neurogenesis	4	1.23E-07
		GO:0048858	cell projection morphogenesis	4	5.27E-07
	**Amy.**		No DEGs at FDR < 0.05		

Hippocampal CEL-induced DEGs were strongly enriched for biological processes and cellular compartments highly relevant to neurodevelopment and behaviour, with the top 3 being: neuron part (GO:0097458; FDR = 3.6E-11), neuron projection (GO:0043005; FDR = 1.4E-09) and locomotor behaviour (GO:0007626; FDR = 5.1E-09). Of the top 20 overrepresented ontologies, fourteen (70%) were specifically neuronal ontologies, four (20%) were related to unspecified cell projection, one (5%) was related to cell signalling and one (5%) behaviour. Of the neuronal ontologies, about half (42.9%) implicated synaptic structure or function. In addition to general neuronal ontologies, the function of many specific or groups of neurotransmitter systems were highlighted in this analysis including: glutamatergic synapse (GO:0098978; FDR = 9.3E-06); catecholamine secretion (GO:0050432; FDR = 1.1E-04); acetylcholine metabolic process (GO:0008291; FDR = 9.7E-04); clathrin-sculpted gamma-aminobutyric acid [GABA] transport vesicle membrane (GO:0061202; FDR = 0.0045); dopamine transport (GO:0015872; FDR = 0.0031). Full gene ontology results for GO levels 2–5 can be found in Supplementary Table 2 in S1 Tables.

#### Cell type enrichment analysis.

In addition to gene ontology analysis, we aimed to investigate which cell types may be particularly susceptible to gene expression changes induced by SI and CEL. EWCE was used to investigate enrichments in DEG sets for marker genes of the brain and nervous system (Zeisel et al. 2018). A total of 265 cell types were analysed in each analysis. Cell types with Bonferroni-corrected p-value of < 0.05 were considered significantly enriched for the DEG sets. Both regions showed one cell type to be enriched for SI-induced DEGs but neither of these cell types were of neuronal origin. Vascular leptomeningeal cells (VLMC2) were significantly enriched for hippocampal DEGs (p < 1e-5) while choroid plexus epithelial cells (CHOR), were significantly enriched for amygdalar DEGs (p < 1e-5).

Six cell types were highlighted as being significantly enriched for CEL-induced hippocampal DEGs. Four of these enriched cell types were forms of medium spiny neuron (MSNs). Two of the enriched MSN subtypes, MSN1 (p < 1E-05) and MSN4 (p < 1E-05) were D1-type, with the other two, MSN2 (p < 1E-05) and MSN3 (p < 1E-05) were D2-type. Two additional cell types were found to be enriched, DEINH2 (p < 1E-05) and DEINH3 (p < 1E-05), both inhibitory neurons of the thalamus. All highlighted cell types used GABA as the main neurotransmitter, indicating DEGs were primarily enriched for marker genes of inhibitory neurons. Full cell type enrichment results can be found in Supplementary Table 3 in S1 Tables.

#### Enrichment for SNP heritability.

The contribution of SNPs at DEG sets to common phenotypes was assessed using sLDSC. Both hippocampal and amygdalar SI-induced DEG sets contained small numbers of total SNPs in the analysis. This was 0.5% for hippocampus and 1.4% for amygdala. CEL-induced DEGs in the hippocampus contained 3.5% of SNPs in the analysis and were enriched for heritability contributing to SCZ, BPD, IQ and EA (Table 2). Neither set showed significant enrichment for heritability for SCZ, BPD, MDD, EA, or IQ. No control phenotypes were significantly enriched. Full sLDSC results can be found in Supplementary Table 4 in S1 Table.

**Table 2:** Enrichment for heritability in psychiatric disorders and cognitive phenotypes in regions surrounding CEL-induced DEGs at FDR < 0.05.

DEG Set	Total SNPs	SCZ		IQ		EA		MDD		BPD	
		**Enrich.**	P*	**Enrich.**	**P**	**Enrich.**	**P**	**Enrich.**	**P**	**Enrich.**	**P**
Hipp.	3.5%	1.68	**0.00082**	1.54	**0.00017**	1.38	**0.0021**	1.03	0.84	1.54	**0.0043**
Amy.	n/a	No DEGs at FDR < 0.05									

*Values in bold survive Bonferroni correction for multiple testing (n = 10 tests including controls)

Enrich. = Fold Enrichment of Heritability; P = p value of enrichment

## Discussion

This paper builds on previous work by our group investigating the behavioural [28] and transcriptomic [22] effects of SI in mouse. Here, we add to this by investigating a new environmental factor and a means of modulating inflammation, chronic celecoxib administration (CEL), in mouse as well as investigating gene expression changes in a new brain region (amygdala). Firstly, to confirm an effect of the SI on behaviour, we investigated the impact of both factors on anxiety-like and social behaviours. Secondly, to confirm an anti-inflammatory effect of celecoxib, we investigated how *ex vivo* cultured splenocytes from these animals respond to TLR stimulation. Finally, we assessed how SI and CEL affect gene expression in the hippocampus and amygdala.

In contrast to [28] where changes were seen in both locomotor and sociability induced by SI, the only behavioural phenotype affected by SI exposure in this work was locomotor activity. This was true for both the OFT, where increased locomotor behaviour was observed and the 3CT, where decreased locomotor was observed. Although in opposite directions, the magnitude of both effects was very similar at ~12.6% increase (OFT) and ~12.5–14.1% decrease (3CT phase 2 and 3). As expected, the administration of CEL *in vivo* reduced cytokine release induced by TLR stimulation in *ex vivo* cultured splenoctyes.

The SI factor had a modest effect on gene expression in both brain regions. Interestingly, we saw inversely correlated expression fold changes between hippocampus and amygdala in this analysis. Both of these regions are involved in fear and anxiety [49] and may be having inhibitory effects on each other in response to SI the stressor. In the hippocampus, GO analysis implicated calcium ion signalling and GABA signalling. Calcium is crucial for neurotransmitter release [50], which may be evidence that inhibitory signalling in particular has been altered by SI here. In humans, disruption of inhibitory GABA signalling is found in many neurodevelopmental and neuropsychiatric disorders, including SCZ [51], ASD [52], MDD [53] and anxiety [54]. Another consistent signal from the GO analysis in SI-induced hippocampal DEGs is insulin growth factor (IGF) binding. In mouse, IGF2 rescued defective hippocampal neurogenesis and cognitive deficits in a model of 22q11.2 Deletion-Associated Schizophrenia [55]. Hippocampal IGF2 has also previously been found to mediate depressive behaviour in rat chronic stress [56]. In human studies, IGF2 was found to be the most strongly downregulated DEG by RNA-seq in the dorsolateral PFC in a cohort of SCZ patients [57]. In the amygdala, the top specific signals implicate neurogenesis, feeding behaviour and axon guidance. Stranahan, Khalil [58] report changes in neurogenesis induced by SI, however, this is in rat hippocampus.

The cell type analysis showed an interesting signature in the SI-induced DEGs, implicating vascular leptomeningeal cells (VLMCs). These cells are at the blood-brain barrier (BBB) interface where endothelium and astrocytes meet [37]. Our previous work [22] implicated populations of astrocytes in a cell type analysis of hippocampal SI-induced DEGs, which support the BBB being affected by SI. Further supporting this is direct evidence in mice that SI affects gene expression of markers of integrity the BBB in the amygdala [59]. Similarly, social stress alters gene expression of integrity markers in the BBB in nucleus accumbens [60] as well as the prefrontal cortex (PFC) [61]. The latter of these studies [61] somewhat replicate these findings in human tissue, showing highly comparable changes in the BBB in post-mortem brain tissue of females diagnosed with MDD [61]. Furthermore, the amygdala showed epithelial cells of the choroid plexus to be enriched for SI-induced DEGs. Interestingly, these cells also form a comparable interface, but this time between cerebrospinal fluid and the brain [37,62].

Although SI appeared to induce relatively comparable magnitude of change in gene expression in both regions, the administration of CEL had a much more heterogeneous response. Here, CEL had no statistically significant effect on gene expression in the amygdala, compared to the hippocampus, where there was a relatively strong response to CEL. The GO analysis revealed a strong effect of CEL exposure on DEGs related to neurogenesis, axon structure and neuron projection. One possible off-target effect for the highly heterogeneous response to CEL may involve the hippocampus’ role in adult neurogenesis [63]. Chesnokova, Pechnick [64] discuss the link between chronic inflammation, neurogenesis and behaviour. There is evidence that specific COX-2 inhibition impacts neurogenesis – including in the mouse hippocampal dentate gyrus via celecoxib [65] and meloxicam [66] and in the mouse subventricular zone using nimesulide [66]. As pointed out in Nam, Kim [65], celecoxib’s effect on angiogenesis could also be having an impact on neurogenesis. The cell type analysis highlighted multiple medium spiny neuron (MSN) populations. MSNs are striatal GABAergic cells and among the most implicated cell types in SCZ [5,67]. Furthermore, the CEL-induced hippocampal DEGs were enriched with common SNP heritability for SCZ, BPD and cognitive ability (IQ, EA), indicating some possible convergence between DEGs dysregulated by CEL administration and common genetic risk for human neuropsychiatric disorders and related traits.

Initially, we would have hypothesised that there would be a bigger difference between the group housed and socially isolated mice in both behaviour and in inflammation. We also expected there be a stronger interaction between the SI and CEL groups. However, there are several limitations to this study. First, based on the limited behavioural effects seen in the OFT and 3CT, we must assume SI had a weak effect on these animals. This is reflected in a small number of DEGs in both brain regions. Findings must be carefully interpreted with the locomotor changes in mind. Second, the cell type analysis must be carefully interpreted as region-specific gene expression may bias results, especially in cell types that are highly specific to one or few regions. Third, although in humans an increased chronic inflammatory response is generally associated with psychiatric disorders, based on studies showing clinical efficacy of CEL, we have investigated the opposite (anti-inflammatory) effect. In conclusion, gene expression changes show convergence with human psychiatric disorders through both enrichments in common genetic heritability and enrichment of previously implicated cell populations.

## Supporting information

S1 Fig Effect of post-weaning Social Isolation (SI) and chronic celecoxib exposure compared to respective group-housed (GH) and vehicle-treated controls on open field test (OFT) velocity. Data are expressed as mean ± SEM.
(TIF)

S2 Fig Effect of post-weaning Social Isolation (SI) and chronic celecoxib exposure compared to respective group-housed (GH) and vehicle-treated controls on three-chambered test (3CT) locomotor activity in A) Habituation B) Sociability and C) Social Memory phases. Data are expressed as mean ± SEM.
(TIF)

S1 Tables Supplementary Table 1. This shows differential expression using log2fold changes between treatment and control groups. Supplementary Table 2. This shows full gene ontology results of DEGs between groups. Supplementary Table 3. This shows significantly enhanced cell types in DEGs between groups. Supplementary Table 4. Shows stratified linkage disequilibrium score regression (sLDSC) investigating SNP heritability in DEGs.
(XLSX)
