# Supplementary figures and images for: Behavioural, immunological and transcriptomic consequences of post-weaning social isolation and chronic celecoxib administration in mouse
Source: PLoS One. 2025 Oct 22;20(10):e0334451. doi: 10.1371/journal.pone.0334451 (PMC12543112; doi:10.1371/journal.pone.0334451)

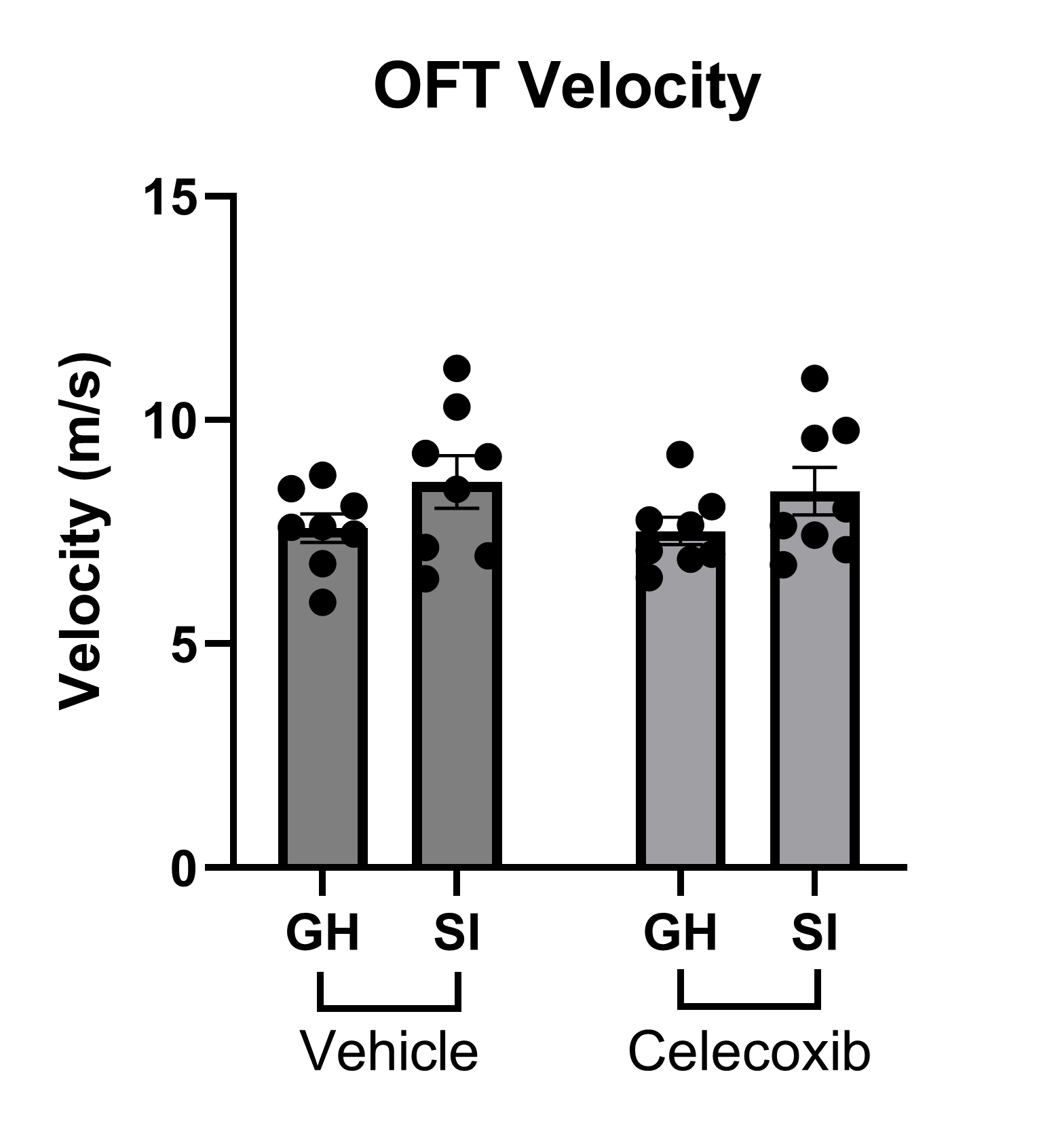

Supplement: S1 Fig — Data are expressed as mean ± SEM. (TIF) [file pone.0334451.s001.tif]

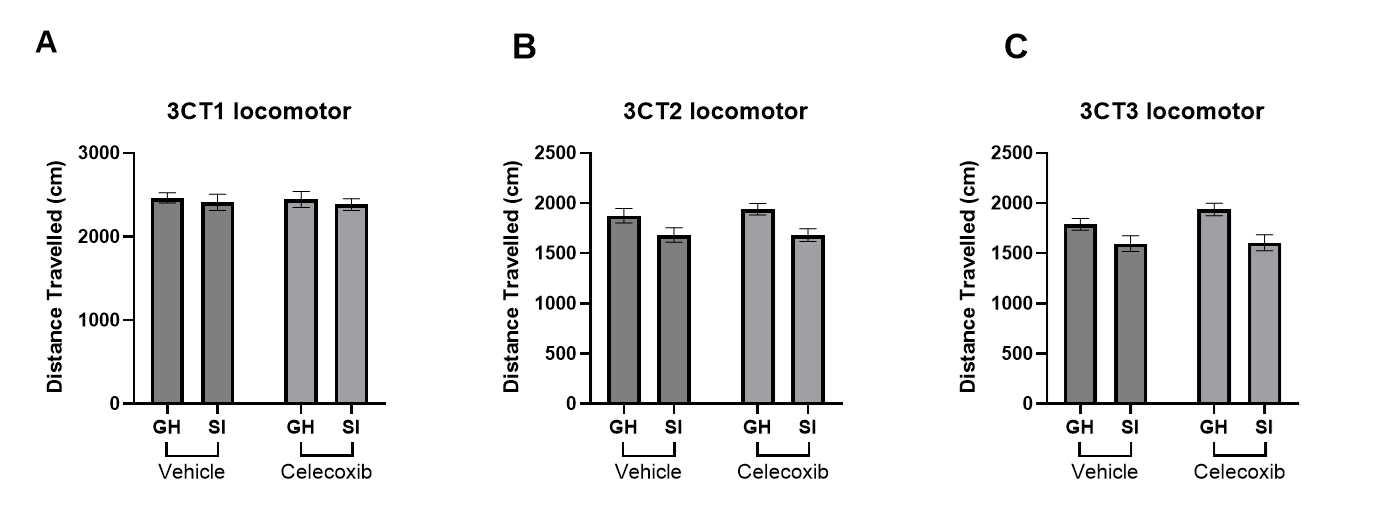

Supplement: S2 Fig — Data are expressed as mean ± SEM. (TIF) [file pone.0334451.s002.tif]
